# Genetic and transcriptomic analyses of diffuse large B-cell lymphoma patients with poor outcomes within two years of diagnosis

**DOI:** 10.1038/s41375-023-02120-7

**Published:** 2023-12-29

**Authors:** Weicheng Ren, Hui Wan, Sulaf Abd Own, Mattias Berglund, Xianhuo Wang, Mingyu Yang, Xiaobo Li, Dongbing Liu, Xiaofei Ye, Kristina Sonnevi, Gunilla Enblad, Rose-Marie Amini, Birgitta Sander, Kui Wu, Huilai Zhang, Björn Engelbrekt Wahlin, Karin E. Smedby, Qiang Pan-Hammarström

**Affiliations:** 1 Division of Immunology, Department of Medical Biochemistry and Biophysics, Karolinska Institutet, Stockholm, Sweden; 2 Division of Clinical Epidemiology, Department of Medicine Solna, Karolinska Institutet, Stockholm, Sweden; 3 Division of Pathology, Department of Laboratory Medicine, Karolinska Institutet and Karolinska University Hospital, Stockholm, Sweden; 4 Department of Immunology, Genetics and Pathology, Uppsala University, Uppsala, Sweden; 5 Department of Lymphoma, Tianjin Medical University Cancer Institute and Hospital, National Clinical Research Center of Cancer, Tianjin’s Clinical Research Center for Cancer, Key Laboratory of Cancer Prevention and Therapy, Tianjin, China; 6 BGI Research, Shenzhen, China; 7 Guangdong Provincial Key Laboratory of Human Disease Genomic, Shenzhen Key Laboratory of Genomics, BGI Research, Shenzhen, China; 8 Kindstar Global Precision Medicine Institute, Wuhan, China; 9 Department of Medicine Huddinge, Karolinska Institutet, Stockholm, Sweden; 10 Department of Hematology, Karolinska University Hospital, Stockholm, Sweden

**Keywords:** Cancer genetics, Cancer genomics, Cancer genomics, Clinical genetics

## Abstract

Despite the improvements in clinical outcomes for DLBCL, a significant proportion of patients still face challenges with refractory/relapsed (R/R) disease after receiving first-line R-CHOP treatment. To further elucidate the underlying mechanism of R/R disease and to develop methods for identifying patients at risk of early disease progression, we integrated clinical, genetic and transcriptomic data derived from 2805 R-CHOP-treated patients from seven independent cohorts. Among these, 887 patients exhibited R/R disease within two years (poor outcome), and 1918 patients remained in remission at two years (good outcome). Our analysis identified four preferentially mutated genes (*TP53, MYD88, SPEN, MYC*) in the untreated (diagnostic) tumor samples from patients with poor outcomes. Furthermore, transcriptomic analysis revealed a distinct gene expression pattern linked to poor outcomes, affecting pathways involved in cell adhesion/migration, T-cell activation/regulation, PI3K, and NF-κB signaling. Moreover, we developed and validated a 24-gene expression score as an independent prognostic predictor for treatment outcomes. This score also demonstrated efficacy in further stratifying high-risk patients when integrated with existing genetic or cell-of-origin subtypes, including the unclassified cases in these models. Finally, based on these findings, we developed an online analysis tool (https://lymphprog.serve.scilifelab.se/app/lymphprog) that can be used for prognostic prediction for DLBCL patients.

## Introduction

DLBCL is one of the most common and aggressive types of lymphoid malignancies, accounting for approximately 30% of all non-Hodgkin lymphomas. Despite its aggressiveness, the disease can be cured, with most patients achieving long-term remission following standard R-CHOP or similar regimens [1, 2]. Nevertheless, approximately one-third of patients experience refractory or relapsed (R/R) disease, and most relapses occur within the first few years [2, 3]. Salvage treatment followed by high-dose chemotherapy and autologous stem cell transplantation has been established as second-line treatment for younger patients with R/R disease, but only a minority of these patients achieve long-term remission [3–6]. Recently, CD19 CAR-T-cell therapy was approved in the US as a second-line treatment in DLBCL patients with R/R disease and as a third-line treatment in several other countries [7, 8]. Other agents also showed promising results, including bispecific antibodies, anti-PD1 antibodies, anti-CD19 antibodies and antibody–drug conjugates [9–11]. Methods for identifying patients who are likely to develop R/R disease within the first few years after diagnosis might benefit from these new therapies and thus are urgently needed to further improve the overall outcomes of DLBCL patients.

Clinically, the International Prognostic Index (IPI) is commonly used to predict disease risk [12, 13]. However, there has been a growing interest in developing more advanced prognostic tools that account for a range of clinical parameters, biomarkers, and gene expression profiling, incorporating immunohistochemical or molecular techniques [14–17]. Twenty years ago, two major subtypes of DLBCL based on cell-of-origin (COO), germinal center B-cell-like (GCB) DLBCL and activated B-cell-like (ABC) DLBCL, were identified by gene expression profiling, with the association of poor outcomes in ABC-DLBCL [18]. In addition, DLBCL patients with a dual rearrangement of *MYC* and *BCL2*, “double/triple-hit” lymphoma, were recognized as a high-grade category with a poor prognosis in the latest WHO and ICC classification [19, 20]. Recently, several studies have identified molecular subtypes associated with different patient outcomes based on genetic alterations (mutations, copy number variation (CNV) and selected translocations) [21–24]. Additionally, direct sequencing of R/R tumors has shown that mutations in several genes (*TP53*, *KMT2D*, *CREBBP*, *NFKBIE*, *FOXO1, MS4A1*) might be associated with therapeutic resistance [25]. However, the prognostic value of these genetic changes remains to be proven. Finally, gene expression signatures [26–29] or tumor cell states and ecosystems [30] based on transcriptomic analysis have been developed and used to predict overall survival (OS) in DLBCL patients. However, to date, there is still a lack of studies that compare or integrate genetic and transcriptomic features when evaluating patient outcomes.

To further understand the mechanism underlying drug resistance and disease progression, and to develop a molecular tool for identifying patients at risk for early R/R disease, here, we performed comprehensive genomic and transcriptomic analyses on diagnostic (untreated) tumors from 2805 R-CHOP-treated DLBCL patients. This included our own study cohort and other six published cohorts with clinical, DNA mutation, and/or gene expression data available [21–24, 31–33]. We characterized the clinical characteristics, mutation profile and gene expression pattern in 887 patients who developed R/R disease within two years and compared these data with those from 1918 patients who remained in remission at two years. Furthermore, we sought to establish a risk classifier capable of effectively predicting the treatment outcomes of DLBCL patients, especially those with early R/R disease.

## Methods

### Clinical and genetic data

This study included analyses of clinical and genetic data from seven DLBCL cohorts. The first cohort (our cohort) included 161 R-CHOP-treated DLBCL patients diagnosed in Sweden or China during 2001–2015 (Table S1A). The Swedish patients (*n* = 73) were diagnosed at Karolinska/Stockholm Country and Uppsala University Hospitals, and their samples were newly sequenced in this study. The Chinese patients (*n* = 88) have been described previously [34–37] and the data were reanalyzed here. DLBCL samples from patients with chronic hepatitis B virus infection were excluded from the current study due to the potential genetic differences observed in these tumors [35]. The performance of various sequencing platforms is summarized in Table S1B, and details of the data analysis, especially on somatic mutation calling and gene expression analysis are provided in the Supplemental Methods. The study was approved by the Institutional Review Boards of Tianjin Medical University Cancer Institute, Uppsala University and Karolinska Institutet.

Six published DLBCL cohorts with available clinical/DNA mutation/gene expression data were also included in the analysis (Table S2A, B) [21–24, 31–33]. Clinical data were collected from the original deposits, and information on LymphGen subtypes was available in five cohorts [21–24, 32, 33]. For the two remaining cohorts (our cohort/GSE117556), LymphGen subtypes were predicted using the LymphGen tool (https://llmpp.nih.gov/lymphgen/index.php).

### Meta-analysis of DLBCL patients with poor and good outcomes treated with R-CHOP

Several criteria were used to enroll samples for meta-analysis: (1) DLBCL patients were treated with R-CHOP and key clinical data were available; (2) Sufficient PFS data were available to define two-year outcomes; (3) Either DNA mutation or gene expression data were available. Subsequently, individuals who experienced R/R disease within two years were characterized as having poor outcomes, whereas those who remained in remission at two years were considered to have good outcomes.

### Differentially expressed genes (DEGs) and gene set enrichment analysis (GSEA)

The RNAseq datasets (our cohort, *n* = 108; Schmitz et al. [23], *n* = 219) and microarray datasets (GSE117556, *n* = 723; GSE181063, *n* = 326) [21, 31] were separately combined using the R package Limma to remove batch effects between datasets [38], generating an RNAseq-based dataset and a microarray-based dataset. DEGs between DLBCLs with poor and good outcomes were identified based on criteria of FDR q < 0.1 and fold change >1.2. Further details on data analysis and GSEA are provided in the Supplemental Methods.

### Establishment and validation of a risk signature to predict R/R disease within two years

The above RNAseq and microarray datasets were further merged into a larger cohort (*n* = 1376), using the quantile normalization approach described previously to normalize cross-platform datasets [39]. Univariate Cox regression was performed to assess the association of PFS and gene expression levels in the entire cohort, identifying prognostic genes (*p* < 0.01). Subsequently, we randomly assigned samples to a discovery cohort (70%, *n* = 964) and a validation cohort (30%, *n* = 412). The LASSO algorithm was used to extract gene-expression risk signatures, using the gene expression levels of overlapping genes between the prognostic genes and the DEGs between the poor and good outcome groups as the input variable and the two-year outcome of each patient as the outcome variable (Fig. S1). In this process, the discovery cohort was further divided into a training cohort (80%, *n* = 772) and a test cohort (20%, *n* = 192). One thousand risk models were constructed by employing various random seeds in the discovery cohort. Subsequently, their performance was evaluated in the test cohort, selecting the model with the highest area under the curve (AUC) value and accuracy for predicting two-year outcomes as the optimal risk model. The validation cohort was then utilized to demonstrate the performance of the risk model. Furthermore, another two independent datasets were used to test the established risk model: validation cohort-2 (RNAseq, *n* = 49) [40], and validation cohort-3 (microarray, remaining sample of GSE181063, *n* = 484). Further details on data computing, risk score calculation, optimal threshold, and risk classification are provided in the Supplemental Methods.

## Results

### Clinical characteristics of different DLBCL cohorts

**Fig. 1:**
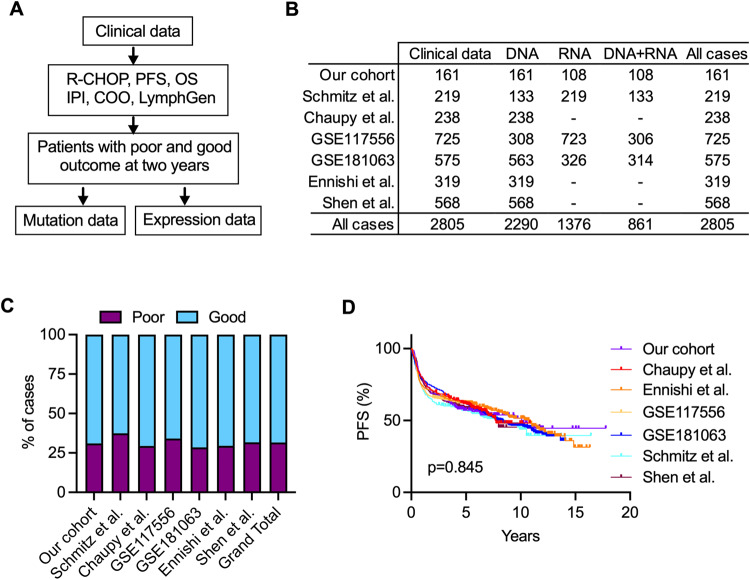
DLBCL cohorts used to identify patients with poor outcomes within two years following R-CHOP treatment. **A** The workflow for identifying patients with poor and good outcomes in different cohorts. **B** An overview of clinical, DNA mutation, and gene expression data for patient cohorts included in this study. **C** The percentage of patients with poor outcomes within each cohort. **D** Kaplan–Meier survival analysis illustrating PFS in different cohorts. The *p* value was calculated by the log-rank test.

To investigate the characteristics of DLBCL patients with early R/R disease following R-CHOP treatment, we initially enrolled 161 DLBCL patients (our cohort; Table S1A). Of these, 50 (31%) experienced R/R disease within two years and were classified as having poor outcomes, whereas the remaining patients who were still in remission at two years, were categorized as having good outcomes. As expected, those with poor outcomes tended to be older, were more likely to have an ABC subtype, and had presented with more advanced disease at diagnosis and higher IPI scores than those with good outcomes (Table S3). To validate these findings, data from six published cohorts were compiled using the same enrollment criteria, comprising 2644 patients (Fig. 1A, B; Table S2A, B). Approximately 32% (range: 29%∼37%) of them experienced R/R disease within two years (Fig. 1C), and these patients displayed similar high-risk characteristics, despite variations in individual parameters among individual cohorts (Table S4). Moreover, the analysis demonstrated no statistically significant difference in PFS among all cohorts, as evidenced by the overlapping survival curves (Fig. 1D). Together, these results suggest that DLBCL patients with poor outcomes share common high-risk characteristics and exhibit similar clinical outcomes across different cohorts. Furthermore, this effort resulted in a cohort of 2805 uniformly R-CHOP-treated DLBCL patients, with 887 of them identified as having poor outcomes within two years. Additionally, we had access to genetic data for 2290 samples and transcriptomic data for 1376 samples (Fig. 1B; Table S2A), with 861 samples having both data types, enabling comprehensive integrated genomic and transcriptomic analyses.

### Mutation spectrum in DLBCL patients with poor outcomes

To investigate the mutation profiles of the 2290 DLBCL tumors, we focused on genes that were sequenced in all cohorts and specifically examined genes that were reported in at least three cohorts. This approach led to the identification of 99 genes for further analysis (Table S5A). Among these, 24 were found to be mutated in at least 10% of DLBCLs, including *KMT2D, PIM1, TP53, BCL2, HIST1H1E, MYD88, CREBBP*, and others (Table S5B). Many of these genes have been previously characterized as significant mutation targets in DLBCL [21–24, 35]. Moreover, the mutation frequencies of these 99 genes in the combined cohort exhibited a strong correlation (Pearson’s correlation coefficients; r value: 0.863–0.963) with those observed in the individual cohorts (Fig. S2A), indicating the consistency of mutation patterns across different cohorts. Furthermore, when examining the mutation frequencies of genes overlapped between the seven cohorts, we also observed a similar consistency (Fig. S2B; Table S5B).

**Fig. 2:**
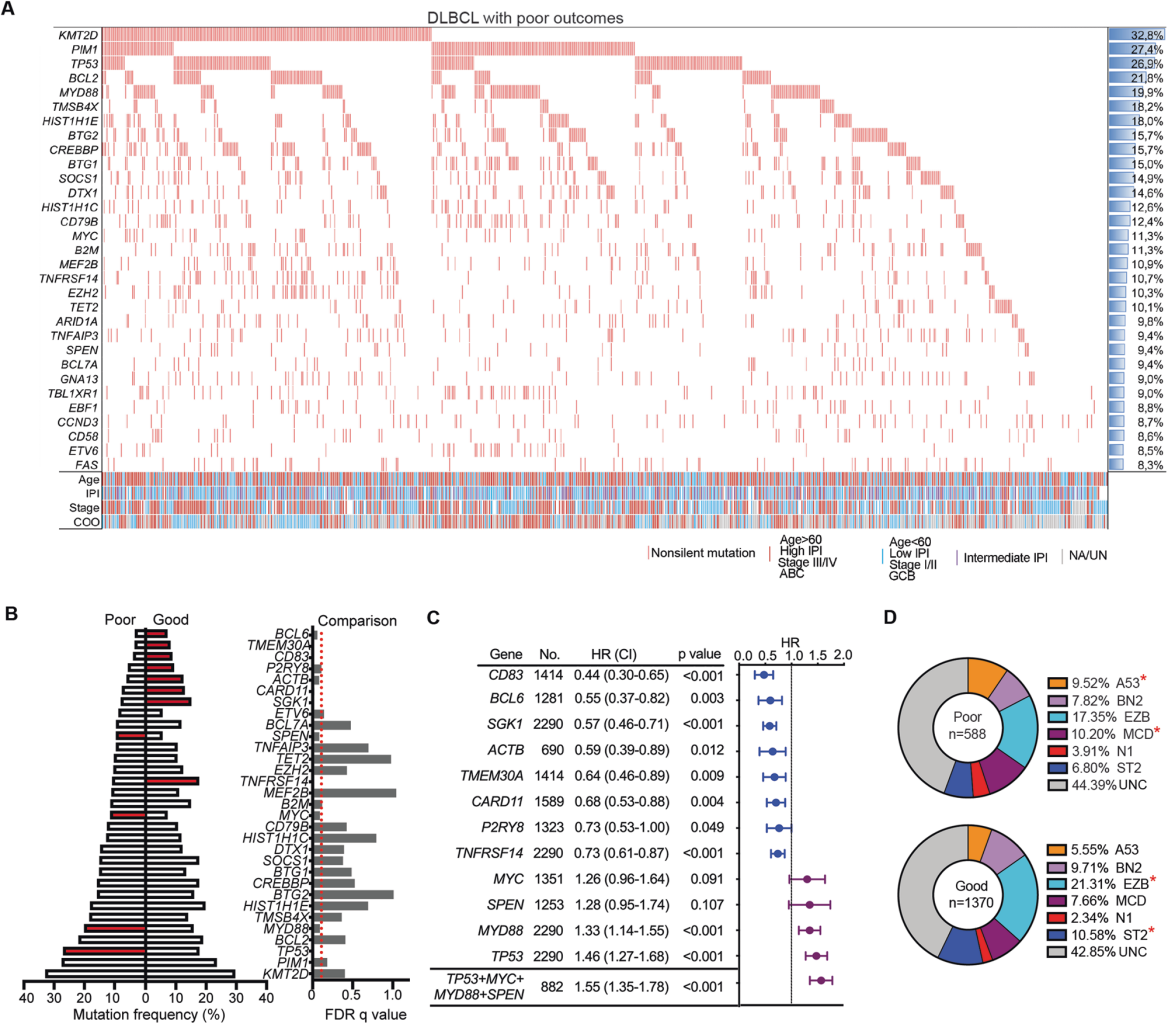
Mutation pattern in tumors derived from DLBCL patients with poor outcomes following R-CHOP treatment. **A** Catalog of the most frequently mutated genes in DLBCL tumors among patients with poor outcomes. Genes that affected by nonsilent mutations across more than three cohorts and observed in more than 8% of all patients (*n* = 702) were included. **B** Comparison of mutation frequencies between DLBCLs with poor and good outcomes using Fisher’s exact test, adjusted by the false discovery rate (FDR) q value (q < 0.1 was considered significant). The presented genes included those most significantly mutated between the two outcome groups and those with mutation frequencies greater than 10%. Genes with FDR q < 0.1 were indicated as red color. **C** Forest plots display the association between the mutation of individual/combined genes and PFS in the combined cohorts. **D** Donut chart illustrating the distribution of LymphGen DNA subtypes in patients with poor or good outcomes in the combined cohorts. Fisher’s exact test was used to compute *p* values. *p < 0.05; NA not available, UN unknown, HR hazard ratio, CI confidence interval, UNC unclassified.

Subsequently, a focused analysis was performed on the mutational profiles of DLBCL patients with poor outcomes in the combined cohort, consisting of 702 patients. We identified 57 genes that were affected by nonsilent mutations in at least 5% of the samples, with 20 of these being mutated in at least 10% of cases (Fig. 2A; Table S6). The most frequently mutated genes in DLBCL with poor outcomes were *KMT2D* (32.8%), *PIM1* (27.4%), *TP53* (26.9%), *BCL2* (21.8%), *MYD88* (19.9%), *TMSB4X* (18.2%), *HIST1H1E* (18.0%), *BTG2* (15.7%), and others. We then compared the mutational profiles of tumors among patients with poor and good outcomes, revealing significant enrichment (q < 0.1) of mutations in four genes (*TP53*, *MYD88*, *SPEN*, *MYC*) and significant depletion of mutations in eight genes (*CD83, BCL6, SGK1, ACTB, TMEM30A, CARD11, P2RY8, TNFRSF14*) in DLBCLs with poor outcomes (Fig. 2B). The examination of these genes within the individual cohorts revealed a largely similar trend in mutation frequency within the poor outcome group across various cohorts (Fig. S3). Moreover, Cox regression analysis demonstrated that mutations in the four enriched genes were associated with worse PFS, whereas mutations in the eight depleted genes were associated with better PFS (Fig. 2C). Combined analysis of mutations in any of the four enriched genes (*TP53* + *MYD88* + *SPEN* + *MYC*) showed a slightly higher hazard ratio than that of individual genes (1.55 vs. 1.26–1.46; Fig. 2C). Finally, approximately 53% of the samples were classified into known LymphGen subtypes, and the A53 and MCD subtypes were significantly overrepresented in patients with poor outcomes, while the EZB and ST2 subtypes were enriched in patients with good outcomes (Fig. 2D). These findings indicate that a distinct mutation spectrum was associated with early R/R disease in DLBCL patients.

### Gene expression profiles in DLBCLs among patients with poor outcomes

**Fig. 3:**
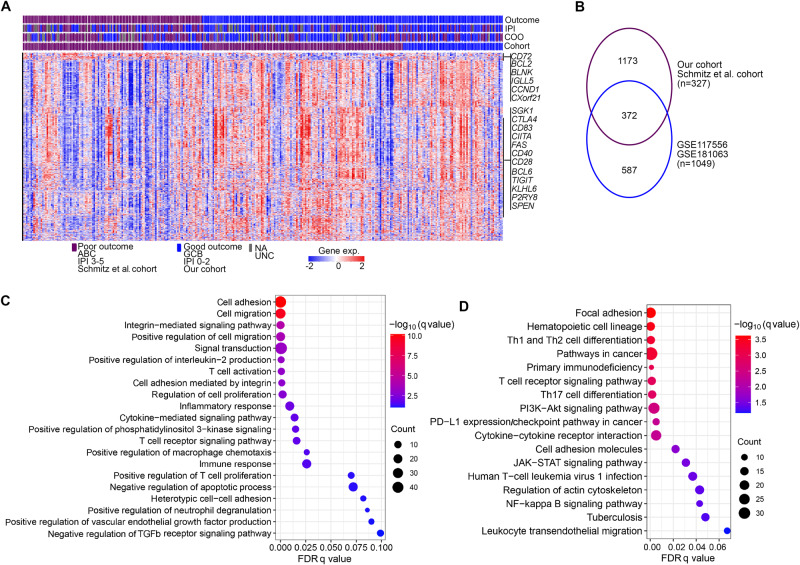
Gene expression pattern in tumors derived from DLBCL patients with poor and good outcomes at two years following R-CHOP treatment. The analysis of differentially expressed genes (DEGs) was conducted separately in the RNAseq dataset (our cohort/Schmitz et al. cohort, *n* = 327) and the microarray dataset (GSE117556/GSE181063; *n* = 1049). A threshold of FDR q < 0.1 and fold change >1.2 was used to define DEGs from each dataset. **A** The heatmap illustrating DEGs between the two outcome groups in the RNAseq dataset. Each column represents a sample, ordered by outcome groups. Within each group, samples were ordered following the input of the data accordingly. Each row represents a gene. Selected genes that are frequently mutated in B-cell lymphomas or important for immune responses are highlighted in the figure. **B** The numbers of overlapping DEGs between the RNAseq dataset and the microarray dataset. GSEA analysis of the overlapping DEGs from (**B**) (**C**: Gene Ontology biological pathway, (**D**): Kyoto Encyclopedia of Genes and Genomes (KEGG) pathway).

To compare the gene expression profiles between good and poor outcome groups, we first conducted analyses of DEGs in the RNAseq dataset (*n* = 327), encompassing 122 cases with poor outcomes (Fig. S4A). In this dataset, DEGs associated with poor outcomes were characterized by a small group of upregulated genes (*n* = 73) that included *BCL2, BLNK, CXorf21, CD72, IGLL5, CCND2, TNFRSF8, NME1, BTLA* and *CD52*, and a large group of downregulated genes (n = 1472) that included 49 genes that have been described to be frequently mutated in lymphoma [35] and immune checkpoint genes (*CTLA4, TIGIT, CD80*) (Fig. 3A; Table S7). Among the DEGs, four genes (*SGK1, CD83, BCL6, P2RY8*) were found to be less frequently mutated in DLBCLs with poor outcomes, whereas one gene (*SPEN*) was preferentially mutated in this group. Subsequently, we analyzed DEGs in the microarray-based dataset (*n* = 1049, Fig. S4B), including 336 cases with poor outcomes, and further compared them with those from the RNAseq-based dataset. This analysis revealed 372 overlapping genes (Fig. 3B), constituting 24% and 39% of the total DEGs in the respective cohorts. Notably, more than 98% (367) of these overlapping DEGs showed consistent upregulation or downregulation in both cohorts (Table S7), suggesting an overall consistency across different datasets and patient groups. We then performed GSEA of the overlapping DEGs and identified several significantly enriched pathways, including cell adhesion/migration/proliferation, PI3K, T-cell activation, T-cell receptor, NF-κB signaling, and PD-1 expression/checkpoint pathways (Fig. 3C, D).

### Establishment and validation of a risk signature to predict early R/R disease

**Fig. 4:**
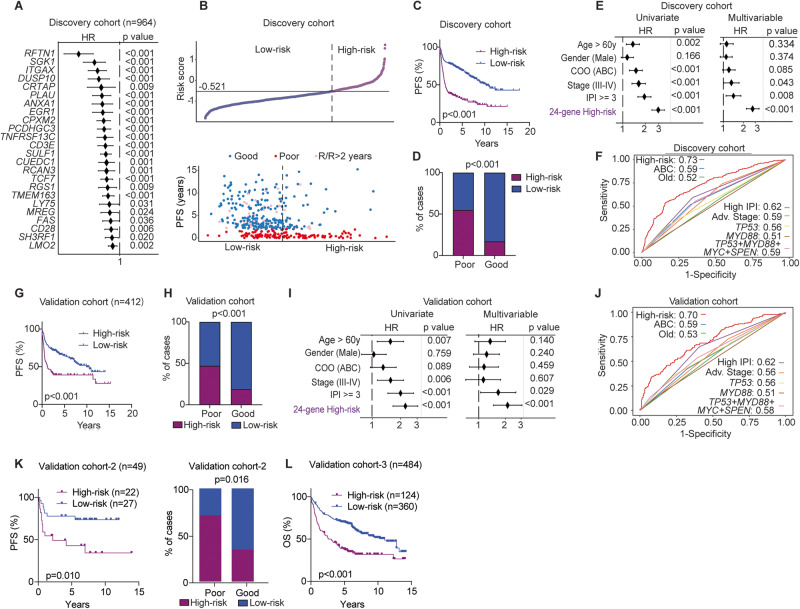
Development and validation of an independent gene-expression signature to predict treatment outcomes in DLBCL patients. The RNAseq and microarray datasets were merged into a larger cohort (*n* = 1376) using a quantile normalization approach. The samples were subsequently randomly divided into a discovery cohort (70%, *n* = 964; **A**–**F**) and a validation cohort (30%, *n* = 412; **G**–**J**). **A** Forest plots showing the association between the expression levels of the 24 genes and PFS within the discovery cohort. **B** The distribution of 24-gene expression scores in each DLBCL patient, and the correlation between PFS and risk groups in the discovery cohort. Patients were assigned to high- and low-risk groups based on the optimal threshold for the ROC curve, set at −0.521. Each dot represents one patient. **C** Kaplan–Meier survival analysis illustrating PFS between high- and low-risk groups in the discovery cohort. The *p* value was calculated by the log-rank test. **D** Bar plots showing the distribution of high- and low-risk patients within poor and good outcome groups in the discovery cohort. Fisher’s exact test was utilized to determine the *p* value. **E** Univariate and multivariable Cox regression analyses demonstrating the prognostic independence of the 24-gene-expression scores in the discovery cohort. Key clinical parameters such as age, subtype, stage, and IPI factors are included in the analysis. **F** ROC curves demonstrating the performance of different parameters in identifying DLBCL patients with two-year poor outcomes in the discovery cohort. AUC values are indicated. **G** Kaplan–Meier survival analysis illustrating PFS of the high- and low-risk groups in the validation cohort (*n* = 412). Patients were classified into high- and low-risk groups using the same threshold established in the discovery cohort (−0.521). The p value was calculated by the log-rank test. **H** Bar plots showing the distribution of high- and low-risk patients within poor and good outcome groups in the validation cohort. Fisher’s exact test was used to determine the *p* value. **I** Univariate and multivariable Cox regression analyses demonstrating the independent prognostic role of the 24-gene expression score in the validation cohort. **J** ROC curves demonstrating the performance of different parameters in identifying DLBCL patients with two-year poor outcomes in the validation cohort. **K**, **L** Two additional independent cohorts (RNAseq=49, CNP0001327; microarray=484, remaining samples of the GSE181063 cohort were only available for OS data) were used to evaluate the algorithm of the 24-gene risk score. HR hazard ratio, CI confidence interval, ROC receiver operating characteristic, AUC area under the curve, OS overall survival.

To establish a robust risk signature representative of early R/R DLBCL, we next employed a quantile normalization approach to integrate RNAseq-based and microarray-based datasets, creating a large, normalized dataset (Fig. S5; *n* = 1376). These samples were then randomly assigned as discovery and validation cohorts (70% and 30% respectively, Fig. S1). Univariate Cox regression was first performed to assess PFS and gene expression levels, identifying 656 prognostic genes from the entire cohort, with 242 of them overlapping with the 372 DEGs (Fig. 3B; Table S8). Using the expression levels of these 242 genes, and the two-year outcomes of each patient as inputs, the LASSO algorithm was subsequently employed to extract risk signatures in the discovery cohort (*n* = 964). Among 1000 established combinations, a 24-gene panel exhibiting the highest AUC value and accuracy in the test cohort in predicting two-year outcomes was selected as the optimal risk model/signature (Fig. 4A; Table S9). Notably, several of these genes play roles in relevant cellular processes/pathways, including BCR signaling (*RFTN1*), protein kinases (*SGK1*), apoptosis (*FAS*), cell differentiation markers (*CD28, CD3E, ITGAX, LY75, TNFRSF13C/BAFFR*), cytokines and growth factors (*PLAU*), and transcription factors (*LMO2, EGR1, TCF7*). Subsequently, using the 24-gene expression score calculated in each tumor, we categorized patients into high- and low-risk groups based on a threshold that provided an optimal trade-off between sensitivity and specificity in predicting two-year outcomes (Fig. 4B). High-risk DLBCL patients assigned by the 24-gene expression score exhibited significantly worse PFS, and more high-risk patients experienced poor outcomes within two years than those assigned to the low-risk group (Fig. 4C, D). Additionally, the 24-gene risk score displayed a positive predictive value (PPV) of 0.61 and a negative predictive value (NPV) of 0.79 in predicting two-year outcomes, with an overall accuracy of 74%. Multivariable analyses revealed that this 24-gene risk score served as an independent predictor (*p* < 0.001) of two-year prognosis, even after adjusting for key clinical risk factors (Fig. 4E). Moreover, ROC curve analysis demonstrated that the 24-gene scoring stratification outperformed the COO subtype, key clinical parameters, and various mutational statuses (*TP53, MYD88*, etc.), in predicting two-year outcomes (Fig. 4F). Furthermore, the inclusion of mutational status of the four preferentially mutated genes (individually or in combination) identified in the poor outcome patients did not enhance its predictive efficiency of early R/R DLBCL (Fig. S6A).

Consistent with the findings in the discovery cohort, we observed worse PFS and more patients experiencing poor outcomes among high-risk patients in the validation cohort (*n* = 412; Fig. 4G, H). The PPV, NPV and overall accuracy in predicting two-year outcomes in the validation cohort were 0.55, 0.75 and 70%, respectively, which were slightly lower than those observed in the discovery cohort. Multivariable analyses and ROC curves confirmed the independent predictive capability of the 24-gene risk score, with an AUC value of 0.7 for predicting two-year outcomes in the validation cohort (Fig. 4I, J). Moreover, combining mutation status (*TP53*, *MYD88*, *SPEN, MYC)* with the 24-gene risk score also failed to improve the prediction performance (Fig. S6B).

Subsequent evaluations, especially among samples with available double-hit and double-expressor status, further confirm its independent prognostic value (Fig. S7). Furthermore, the comparison of ROC curves with existing gene expression-based classifiers [27, 28, 41] demonstrated that our 24-gene risk score had the best performance in predicting two-year outcomes (Fig. S8). In addition, the results across individual cohorts revealed similar patterns in 24-gene expression, differences in PFS, and the distribution of patients in high- and low-risk groups (Fig. S9–10). Finally, the 24-gene score algorithm was successfully validated on two additional cohorts derived from different platforms (RNAseq, *n* = 49; microarray, *n* = 484) (Fig. 4K–L). These findings underscore the robustness and generalizability of the 24-gene expression score as an independent prognostic tool for predicting early disease progression in DLBCL.

### The association of the 24-gene expression score and COO subtypes of DLBCL

**Fig. 5:**
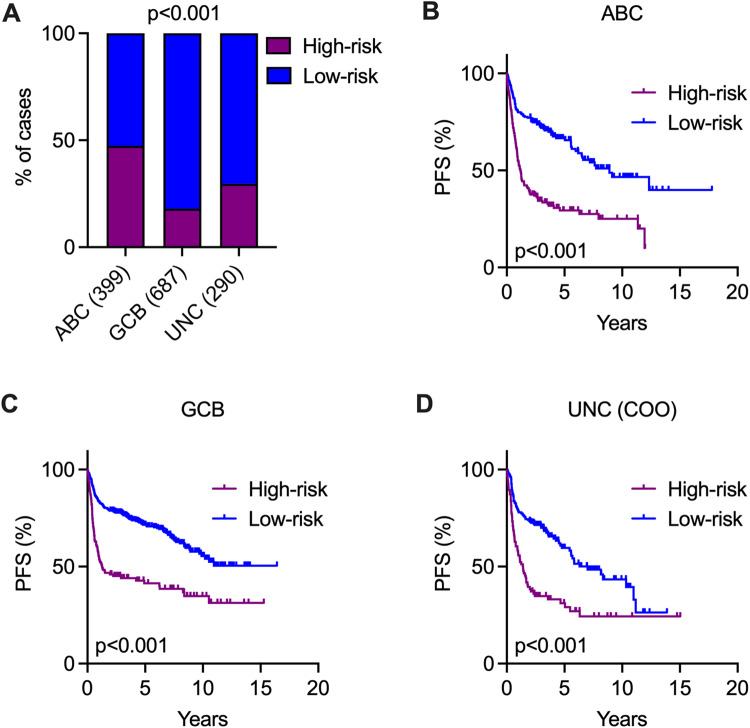
Independent risk stratification by 24-gene expression scores in various COO subtypes of DLBCLs. The analysis was performed on all samples with available gene expression data (*n* = 1376). **A** Bar plots showing the distribution of high- and low-risk patients in various COO subtypes. Fisher’s exact test was used to determine the *p* value. Kaplan–Meier survival analysis illustrating PFS between high- and low-risk groups in ABC-DLBCL (**B**), GCB-DLBCL (**C**) and UNC-DLBCL (**D**). COO cell-of-origin, UNC unclassified. The *p* value was calculated by the log-rank test. Independent analyses in the individual cohorts, discovery cohort and validation cohort are presented in Fig. S11–S12.

We next assessed the correlation between the 24-gene expression score and the COO subtypes, which are known predictors of DLBCL patient outcomes. Within the entire cohort (*n* = 1376), a notably higher proportion of ABC-DLBCL cases were categorized as high-risk, while a lower percentage of GCB-DLBCL and unclassified cases fell into the high-risk group (Fig. 5A). Moreover, patients classified as high-risk exhibited significantly poorer PFS in both ABC and GCB subtypes when compared to those in the low-risk group (Fig. 5B, C). Notably, the 24-gene risk score was also effective in identifying high-risk patients among the unclassified COO cases (Fig. 5D). Furthermore, these results were consistent across the discovery, validation, and individual cohorts, indicating no significant cohort-related bias in the analysis of combined data (Fig. S11–12). Together, our findings suggest that the 24-gene expression score can further stratify high-risk patients among COO subtypes, including those unclassified cases.

### Use the 24-gene expression score to stratify high-risk patients within LymphGen subtypes

**Fig. 6:**
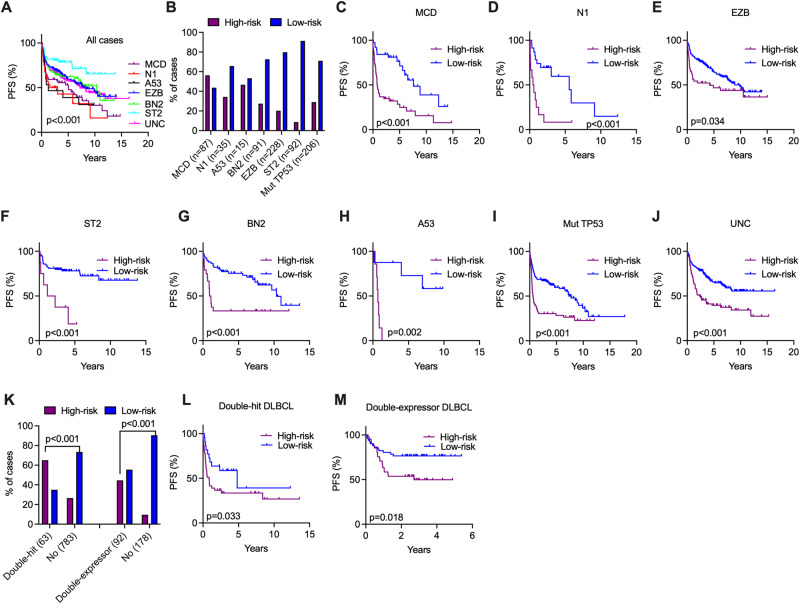
Individualized risk stratification by 24-gene expression score in LymphGen DNA subtypes. All samples with available gene expression data and LymphGen DNA subtypes were combined for the analysis (*n* = 956). **A** Kaplan–Meier survival analysis showing the PFS in the individual LymphGen DNA subtypes. **B** Bar plots showing the distribution of high- and low-risk patients in the different DNA subtypes. **C**–**J** Kaplan–Meier survival analysis showing the PFS of high- and low-risk patients in the indicated DNA subtypes and the unclassified subtype. **K** Bar plots showing the distribution of high- and low-risk patients in the double-hits (*n* = 63) and double-expressors (*n* = 92) of MYC and BCL2. Among 1376 samples with 24-gene risk scores, 846 cases were evaluated for double-hit status, with 63 identified as double-hit. A total of 270 samples were assessed for double-expressor status, with 92 identified as double-expressors. Fisher’s exact test was used to compute *p* values. **L**, **M** Kaplan–Meier survival analysis showing the PFS of high- and low-risk patients in the indicated groups. UNC unclassified. For all Kaplan–Meier survival analyses, the *p* value was calculated by the log-rank test. Independent analyses in the discovery and validation cohorts are presented in Fig. S13.

To investigate the potential relationship between the 24-gene expression score and DNA molecular subtypes in DLBCLs, we examined the performance of the risk scores across LymphGen subtypes. Among the 1376 samples with transcriptomic data, LymphGen subtype information was available for 956 samples. Consistent with previous studies [23, 24], the MCD, N1, and A53 subtypes in the entire cohort were associated with inferior survival, whereas the EZB, BN2, and ST2 subtypes had favorable outcomes (Fig. 6A). The distribution of high- and low-risk patients in these DNA subtypes is summarized in Fig. 6B and the 24-gene risk score was significantly effective in further stratifying high-risk patients across all individual DNA subtypes (Fig. 6C–H). Since the A53 subtype of LymphGen is mainly defined by *TP53* mutations and CNVs and since CNV data were not available in three cohorts (our cohort/GSE117556/GSE181063), we conducted an independent analysis on DLBCLs with *TP53* mutations (*n* = 206). Notably, patients carrying *TP53* mutations who were assigned to the high-risk group had significantly worse PFS than those assigned to the low-risk group (Fig. 6I). Moreover, in those 284 unclassified cases, where LymphGen subtypes were not assigned and *TP53* were not mutated (Table S2A), the 24-gene risk score was still able to effectively stratify high-risk patients (Fig. 6J). Furthermore, the 24-gene risk score also demonstrated its significant capability in assigning double-hits and double-expressors into the high-risk group (Fig. 6K), and further identifying high-risk patients within each subtype (Fig. 6L–M). Additionally, these analyses were conducted separately in the discovery and validation cohorts, and the results revealed relatively consistent results observed in the combined cohort (Fig. S13). Thus, the utility of the 24-gene expression score can be extended to risk stratification among the genetic subtypes.

## Discussion

Despite recent advances in the characterization of the genome and transcriptome of DLBCL [21–24, 32–36, 42–46], including analysis at the single-cell level [44], our knowledge of patients with early R/R disease is still limited. Our study integrated data from seven cohorts comprising 2805 R-CHOP-treated patients, including 887 cases with poor treatment outcomes within two years. Our analyses revealed a distinct mutation pattern and gene expression profile in these patients with poor outcomes. Additionally, we established a 24-gene expression score as an independent prognostic predictor for early R/R disease in DLBCL patients. This risk score also demonstrated its effectiveness in further stratifying COO and genetic subtypes into high- and low-risk groups. Overall, our study provides valuable insights into understanding the molecular mechanisms underlying early R/R DLBCL and highlights the importance of incorporating gene expression profiling into clinical decision-making for DLBCL patients.

Various studies have previously utilized NGS technologies to describe genetic subtypes and prognostic models in DLBCL, including DNA mutation-based subtypes [21–24] and gene expression-based risk signatures [26–29] or prognostic tumor microenvironments [30]. However, the focus has been largely on OS, rather than specifically addressing features associated with early disease progression. Some studies have developed risk models by incorporating OS data from patients treated with CHOP and R-CHOP [27, 28], whereas some risk signatures were developed without validation cohorts or based on limited sample sizes [26, 29]. Moreover, none of these risk models/signatures attempted to integrate both genetic and transcriptomic data in their evaluation. Our study benefits from many patients with both genomic and transcriptomic data and the use of multiple independent cohorts to validate findings. Moreover, we defined early R/R disease based on a two-year cutoff, which is associated with the poorest outcomes [6, 47]. Notably, previous studies indicated that patients who survived and remained event-free for 24 months had a similar consecutive OS as the general population matched for sex and age [6, 48]. Our newly developed 24-gene classifier is primarily designed to optimize its effectiveness in predicting two-year outcomes, surpassing the performance of existing classifiers in this aspect. Moreover, the integration of the cross-platform normalization approach facilitates the merging of data from diverse platforms, generating a robust dataset for establishing a more reliable risk classifier. Finally, in light of our findings, we created an online analysis tool (accessible at https://lymphprog.serve.scilifelab.se/app/lymphprog) designed for user-friendly and efficient risk assessment, which lays the foundation for developing a practical algorithm with potential applications in clinical settings.

Four genes were preferentially mutated in DLBCL with poor outcomes. Of these, *TP53, MYC*, and *MYD88* mutations have been associated with early R/R disease in DLBCLs in previous studies [25, 49–52], whereas *SPEN* is a novel candidate, and plays a role in negatively regulating NOTCH signaling. Studies have indicated that aberrant NOTCH signaling can contribute to drug resistance in various cancers [53], including DLBCL [54], suggesting that targeting the NOTCH pathway may hold promise as a therapeutic strategy for patients with early R/R disease. Conversely, the eight genes preferentially mutated in DLBCL with good outcomes align with their roles as markers for genetic subtypes associated with favorable prognosis, including BN2, ST2, and EZB [23, 24]. Loss-of-function *TMEM30A* mutations have been associated with increased B-cell signaling and enhanced drug uptake in DLBCLs, potentially contributing to a better treatment response [55]. Notably, the study also identified genes, such as *SGK1* and *CD83*, where both higher mutation frequency and higher mRNA expression were linked to better treatment outcomes in DLBCL patients. This suggests that tumor cells with such features may exhibit increased sensitivity to R-CHOP treatment. However, although the identification of these differentially mutated genes may help to understand the mechanism underlying early R/R disease, the mutation status of individual genes appears to provide limited predictive value when compared to gene expression-based risk signatures.

We further demonstrated that our risk score algorithm shows superior performance in further stratifying high-risk patients from COO and DNA molecular subtypes. Importantly, it showed the capacity to further identify high-risk patients among the unclassified cases in these subtyping tools. While this 24-gene risk score may not identify all high-risk patients, possibly due to the considerable intratumor and intertumor heterogeneity of the disease, it shows promise in effectively predicting two-year outcomes in more than 70% of DLBCL patients following R-CHOP treatment. For high-risk patients identified by this classifier, intensified immunochemotherapy protocols, such as incorporating high-dose methotrexate [56], or R-double-CHOP [57], may be considered while awaiting more definitive evidence on new immunotherapies or combined therapies [58]. Moreover, close monitoring of high-risk patients during or after R-CHOP treatment is necessary. Tools such as monitoring measurable residual disease through cell-free tumor DNA analysis may help to track treatment response and disease status in these high-risk patients [59], facilitating timely interventions if needed.

In summary, leveraging data from multiple cohorts, our study identified mutated genes, altered pathways, and gene expression-based risk signatures associated with poor outcomes in DLBCL patients. However, despite combining several cohorts to achieve a relatively homogeneous dataset, considerable heterogeneities (sample resources, sequencing platforms/methods, variant callers, thresholds in identifying prognostic genes and DEGs) between acquired data types from individual cohorts may still introduce variability. Therefore, these findings may require further validation in additional cohorts. To further improve the predictive value of the 24-gene expression score, incorporating additional genetic/epigenetic features, such as genome-wide mutational signatures [37], CNVs, noncoding drivers [60], RNA editing [46], and tumor microenvironment, as well as the application of supplementary tools, such as proteomic, metabolic, and single-cell studies, may be considered.

### Supplementary information

Supplemental Methods and Figures

Supplemental Tables

## Data Availability

The datasets analyzed during the current study are available in the China National GeneBank Sequence Archive of China National GeneBank Database with accession Nos. CNP0001228 and CNP0001220, and in the National Center for Biotechnology Information (NCBI) database with accession No. PRJNA952204.
