# Supplementary material for: Genetic and transcriptomic analyses of diffuse large B-cell lymphoma patients with poor outcomes within two years of diagnosis
Source: Leukemia. 2023 Dec 29;38(3):610–20. doi: 10.1038/s41375-023-02120-7 (PMC10912034; doi:10.1038/s41375-023-02120-7)
Supplement: Supplementary file 1 — Supplemental Methods and Figures [file 41375_2023_2120_MOESM1_ESM.pdf]

MATERIALS AND METHODS

Sample information

For our cohort, all samples were diagnosed before the 2016 WHO classification, and translocations involving *MYC* and *BCL2/BCL6* were not routinely investigated at diagnosis. Informed consent was obtained from all patients. All samples (seven cohorts) included in this study were *de novo* DLBCL and patients with transformed disease were excluded. Patients with immunodeficiency (HIV) were excluded from our cohort, Ennishi et al. cohort, Chaupy et al. cohort, and GSE117556 cohort, whereas this information in the remaining cohorts (Schmitz et.al, GSE181063, Shen et.al. cohorts) was not mentioned in the original references.

Among 1376 samples with gene expression data, 846 cases were evaluated for double-hit status, with 63 identified as double-hit. A total of 270 samples were assessed for double-expressor status, with 92 identified as double-expressors. Double-hit status was accessible in a subset of all four cohorts with gene expression data, while double-expressor status was available solely in the GSE117556 cohort. All the information is summarized in Table.S2A.

Whole genome/exome sequencing (WGS/WES) and targeted sequencing

A DNeasy Tissue and Blood Kit (Qiagen) was used to extract DNA. WGS and WES were performed at Macrogen Europe using either the HiSeq 2000 or HiSeq X10 platforms (Illumina). The targeted sequencing panel (lymphochip) included 212 genes that were frequently mutated in different types of B-cell lymphomas, important for DNA repair, or important for targeted therapy[1]. For the Swedish cohort, 43 pairs of DLBCL tumors with matched peripheral blood samples were sequenced by WES and 30 tumor-only samples were tested by lymphochip (Table.S1A). The sequencing data for the 88 Chinese DLBCLs have been described

previously[1-4] and reanalyzed here, including 25 and 15 paired samples sequenced by WGS or WES, respectively, and 48 tumor-only samples sequenced by lymphochip.

Burrows–Wheeler Aligner software was used to align reads to the human reference genome[5]. For the identification of somatic mutations from paired tumors/controls, single-nucleotide variants (SNVs) were detected using VarScan[6], whereas somatic insertions and deletions (InDels) were identified by GATK (WES)[7] or Platypus (WGS)[8]. All reported SNV and InDels passed visual inspection using Integrative Genomics Viewer[9]. For the identification of mutations from tumor-only samples, VarScan was used to detect SNVs and InDels using the defined parameters, followed by several steps of filtering to remove potential germline mutations[1].

Transcriptome sequencing and analysis

Transcriptome sequencing was performed on 108 available tumors (Swedish patient: n=49; Chinese patients: n=59). TRIzol (Invitrogen) was used to extract total RNA from tumor samples. For the Swedish cohort, the libraries were prepared at Macrogen Europe following the manufacturer's instructions and sequenced on a HiSeq 2000 platform. For the Chinese cohort, the libraries were sequenced on a HiSeq 2000 platform, and the details of sequencing and data analysis have been described previously[1]. The transcripts per million (TPM) was used to determine gene expression levels, and Log2-transformed TPM values were normalized by Limma to remove batch effects[10]. The normalized expression values were analyzed by Qlucore Omics Explorer (Qlucore AB, Lund, Sweden).

Differentially expressed genes (DEGs) and gene set enrichment analysis (GSEA)

To identify DEGs between DLBCLs with poor and good outcomes, the normalized gene expression data were analyzed using Qlucore Omics Explorer (Lund, Sweden), and genes with a false discovery rate (FDR)-adjusted p value (q-value hereafter) <0.1 and fold change >1.2 between groups were considered DEGs. GSEA of DEGs was performed using KEGG and Gene Ontology pathways in the DAVID tool (https://david.ncifcrf.gov/)[11]. Pathways with a q-value <0.1 were considered significantly enriched.

Establishment and validation of a risk signature to predict R/R disease within two years

The RNAseq (n=327) and microarray (n=1049) datasets used in the DEG analysis were further merged into a larger cohort (n=1376), using the quantile normalization approach described previously to normalize cross-platform datasets[12]. After data normalization, the establishment and validation of risk signatures were performed as follows:

1). Univariate Cox regression was performed to assess the association between gene expression levels and PFS in the entire cohort. A total of 656 genes (referred to as prognostic genes) were identified with a prognostic significance (p values < 0.01).

2). We randomly divided the samples into a discovery cohort (70%, n=964) and a validation cohort (30%, n=412). In the discovery cohort, we utilized the least absolute shrinkage and selection operator (LASSO) logistic regression to extract a prognostic signature for predicting two-year outcomes. This involved using the expression level of 242 overlapping prognostic genes and the DEGs (overlapped DEGs from the RNAseq and microarray-based datasets) as inputs and the two-year outcome of each patient as the outcome variable. In this process, several steps were undertaken: 1) The discovery cohort was further randomly divided into training (80%) and test cohorts (20%). 2) Utilizing data from the training cohort, we employed the LASSO algorithm to construct a model for predicting two-year outcomes, employing a 10-fold cross-validation approach. 3) Various parameters were used to measure the performance of the

established model using the test cohort, including the receiver operating characteristic (ROC) curve, area under the curve (AUC), accuracy, sensitivity, and specificity. Subsequently, we repeated steps 1-3 using random seeds to establish 1000 different risk models. Finally, the model exhibiting the highest AUC value and accuracy rate in predicting two-year outcomes within the test cohort was selected as the optimal risk model.

3). The risk score of each patient was calculated using the following formula: risk score= $\sum_{i=1}^{n} Coef_i * x_i + Intercept,$ where $Coef$ (coefficient factor) and Intercept were identified in the LASSO algorithm and x_i is the expression value of each gene.

4). We determined the optimal threshold of the risk model using the following approach: In the discovery cohort, we employed the optimal threshold approach to classify high- and low-risk groups. In this approach, all risk scores were used as predictors, and the two-year outcomes were used as responses. These data were used as input in the following analysis. Using all risk scores, we utilized the mean values of each adjacent risk score to set up a range of testing thresholds. For each testing threshold, we calculated the corresponding sensitivity and specificity in identifying two-year outcomes and then created an ROC curve by plotting sensitivity against specificity. We identified the optimal threshold as the one with the maximum Youden's J statistic, providing the best balance between sensitivity and specificity for risk classification of two-year outcomes. This same threshold was subsequently used in the validation cohorts to stratify patients. These analyses were performed using various R packages, including Survival (V3.3.1), Glmnet (V4.1.4), and pROC (V1.18.0).

5). The performance of the optimal model was subsequently validated in the validation cohort (n=412).

6). Based on the computational procedures and optimal gene-expression risk scores, we developed an online version for risk prediction of DLBCL patients treated with R-CHOP, which is available at https://lymphprog.serve.scilifelab.se/app/lymphprog. Moreover, we conducted

additional testing of the algorithm using another two independent cohorts: one with RNAseq data (Validation cohort-2) consisting of 49 samples (CNP0001327[13]) and another with microarray data comprising 484 samples (Validation cohort-3), which were part of the GSE181063 cohort but had only OS data available. These validation datasets were derived from various platforms and were completely independent of those employed in the discovery and validation phases.

Statistics

P values were calculated using the $\chi 2$-test, Fisher's exact test, or the Mann–Whitney U test (two-tailed). Statistical tests were performed with SPSS or GraphPad Prism 9 or R 4.3.0.

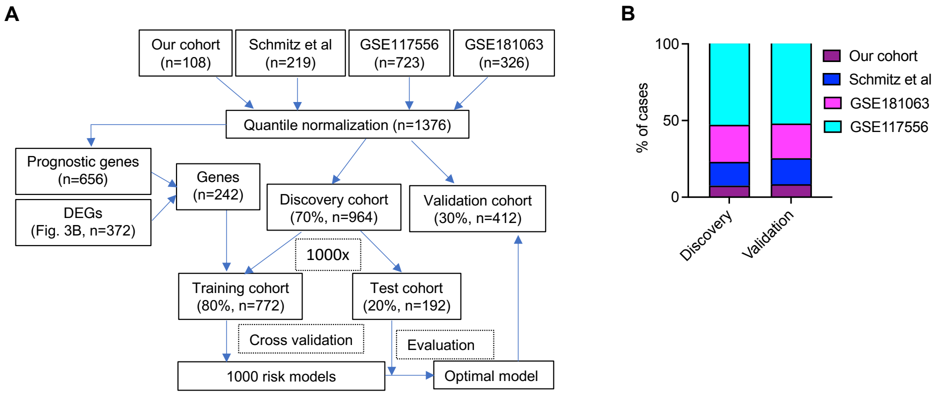

Figure S1. Flow chart of the establishment and validation of a risk signature in predicting two-year outcomes in DLBCL patients treated with R-CHOP.

(**A**) An outline of the process for developing and validating risk signatures. (**B**) Bar plots showing the proportional distribution of samples from individual datasets within the discovery and validation cohorts.

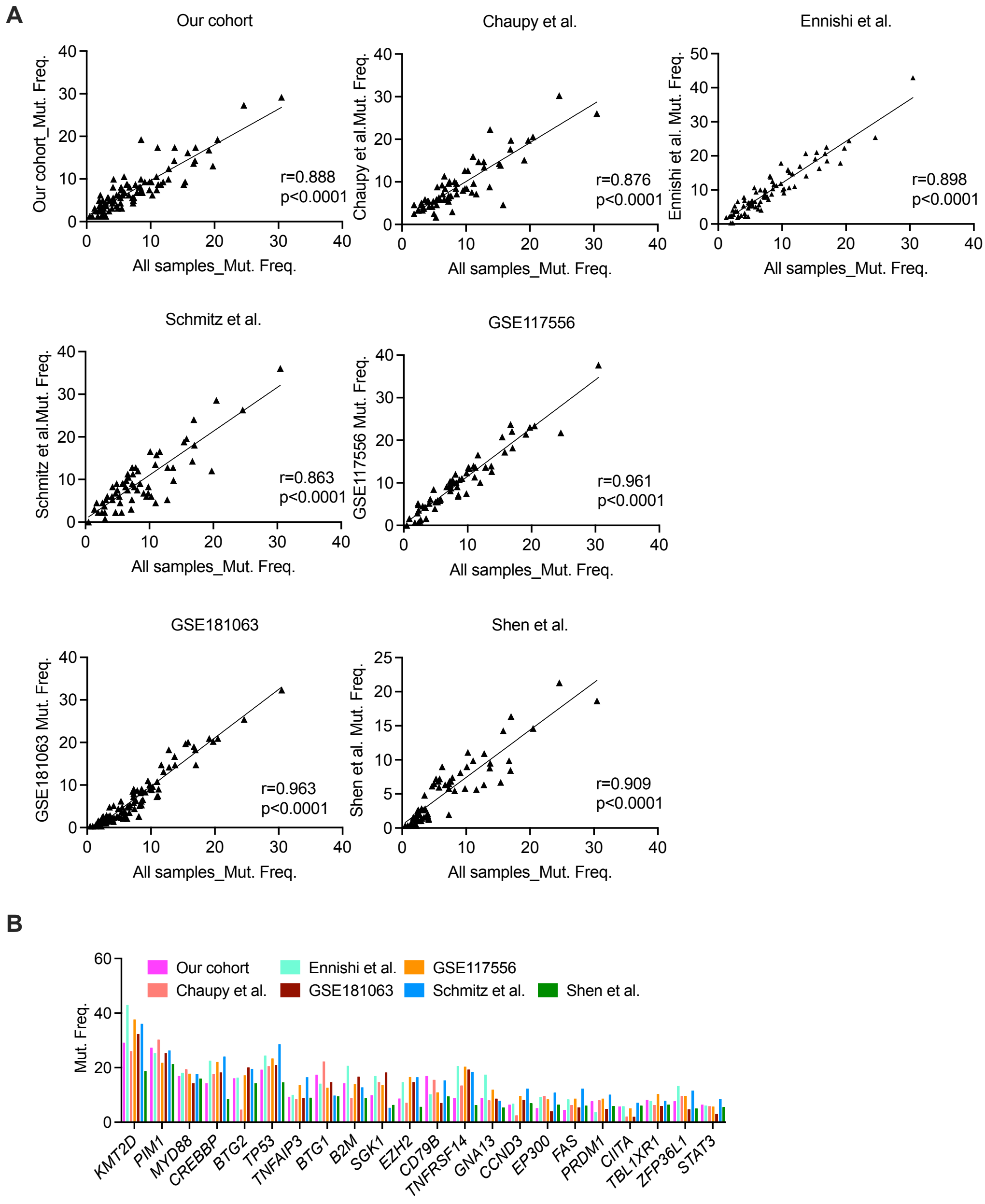

Figure S2. Correlation analysis of gene mutation frequencies between the combined cohort and individual cohorts.

(**A**) The scatter plots display the correlation in gene mutation frequencies between individual cohorts and the combined cohort (All samples). The Pearson correlation coefficient was used to analyze the r values. (**B**) The dodged bars illustrate the mutation frequencies of key mutated genes across individual cohorts. Mut, mutation. Freq, frequency.

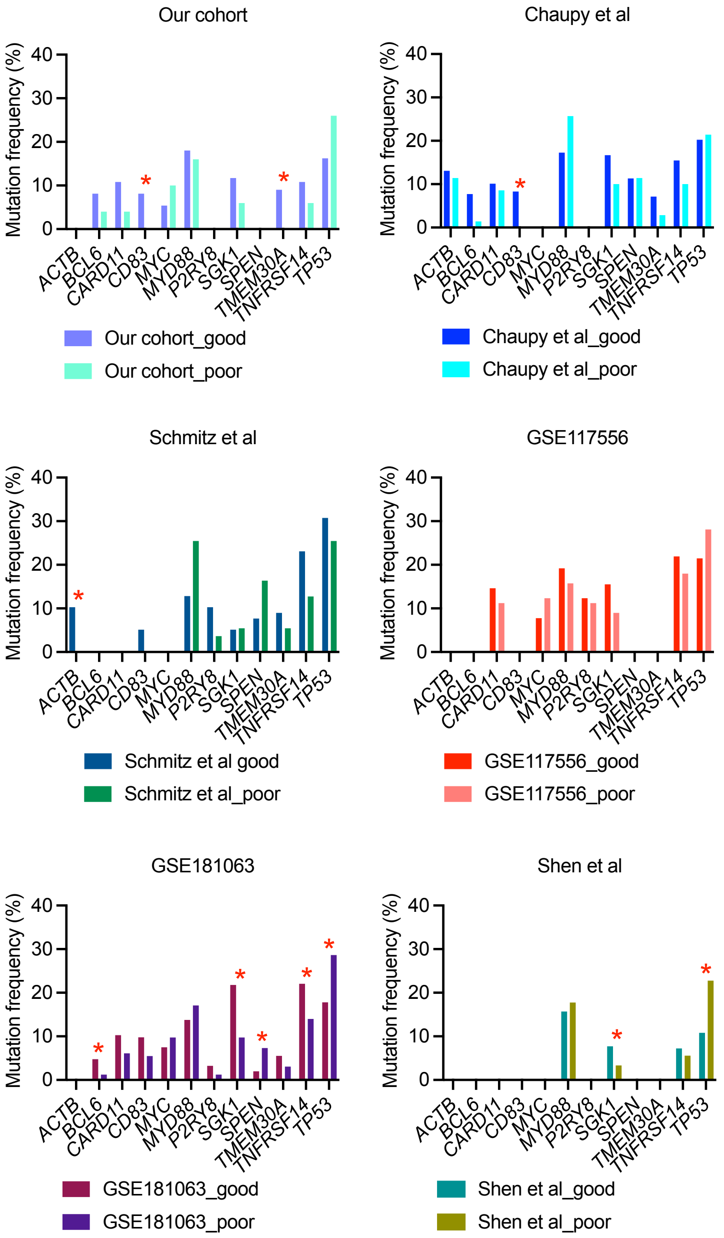

Figure S3. Comparison of mutation frequencies of significantly mutated genes in poor and good outcome groups from individual cohorts.

The bar charts display the mutation frequencies of the 12 genes presented in Figure 2C in individual cohorts. The chi-square test was used to calculate *p* values. *, $p<0.05$.

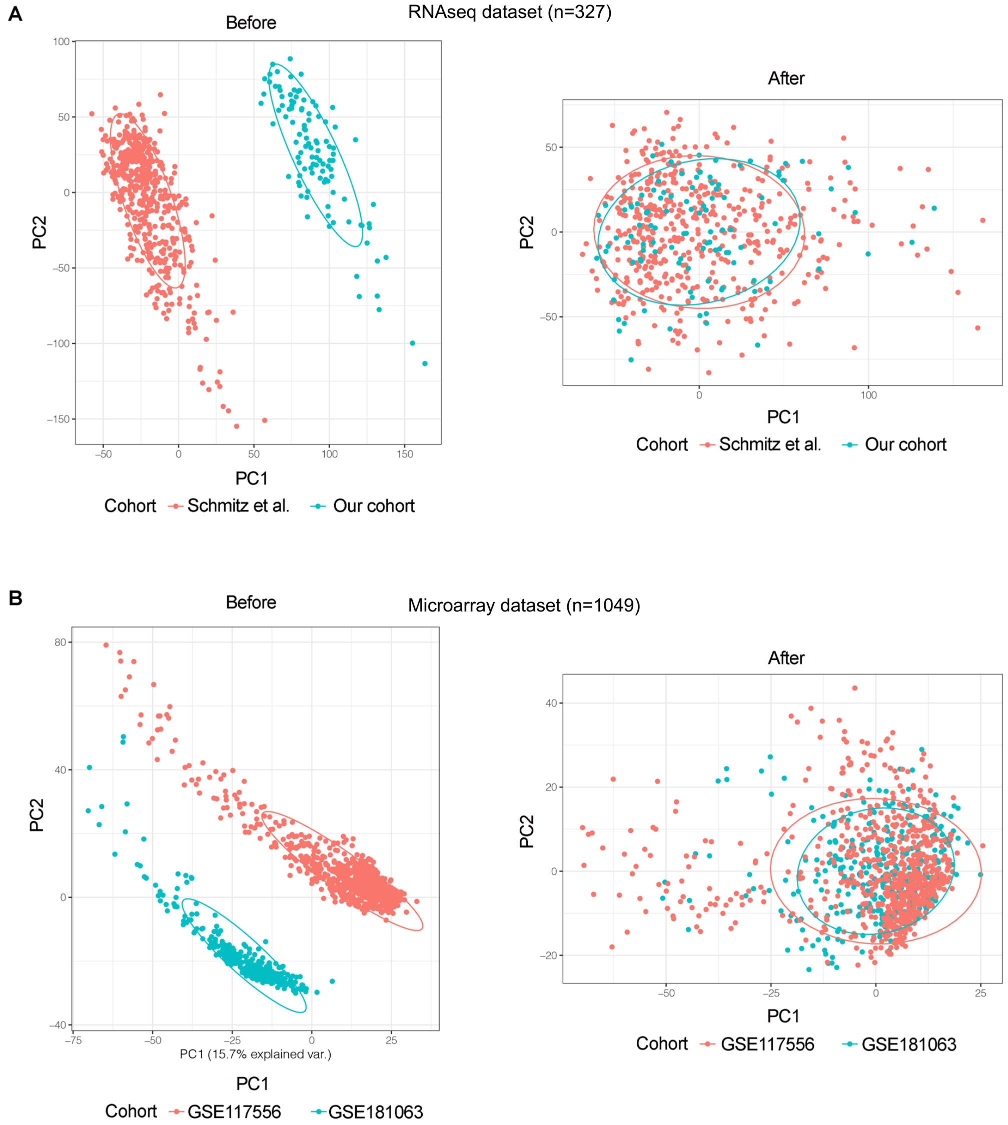

Figure S4. Establishment of two datasets for identifying differentially expressed genes. Gene expression data from four cohorts were analyzed, comprising (**A**) two RNAseq datasets (our cohort, n=108; Schmitz et.al., n=219) and (**B**) two microarray datasets (GSE117556, n=723; GSE181063, n=326). To remove batch effects between datasets, the RNAseq and microarray datasets were merged separately using the R package Limma. This resulted in two combined datasets, an RNAseq-based dataset (n=327) and a microarray-based dataset (n=1049).

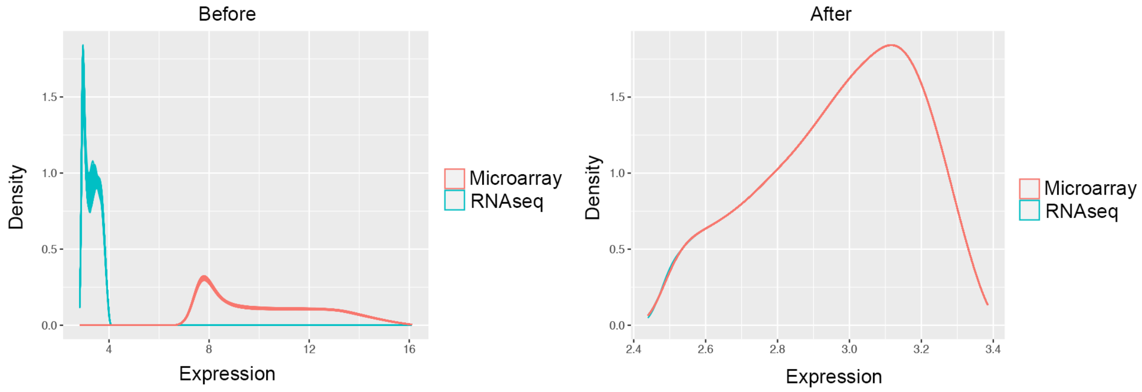

Figure S5. Cross-platform normalization of gene expression data using quantile normalization approach.

To establish a risk model that generalizes well, we combined RNAseq (n=327) and microarray (n=1049) datasets into a larger dataset (n=1376), using the quantile normalization approach described previously[12], which showed good performance for cross-platform normalization. The graphs illustrate the data distribution from RNA-seq and microarray datasets before and after quantile normalization.

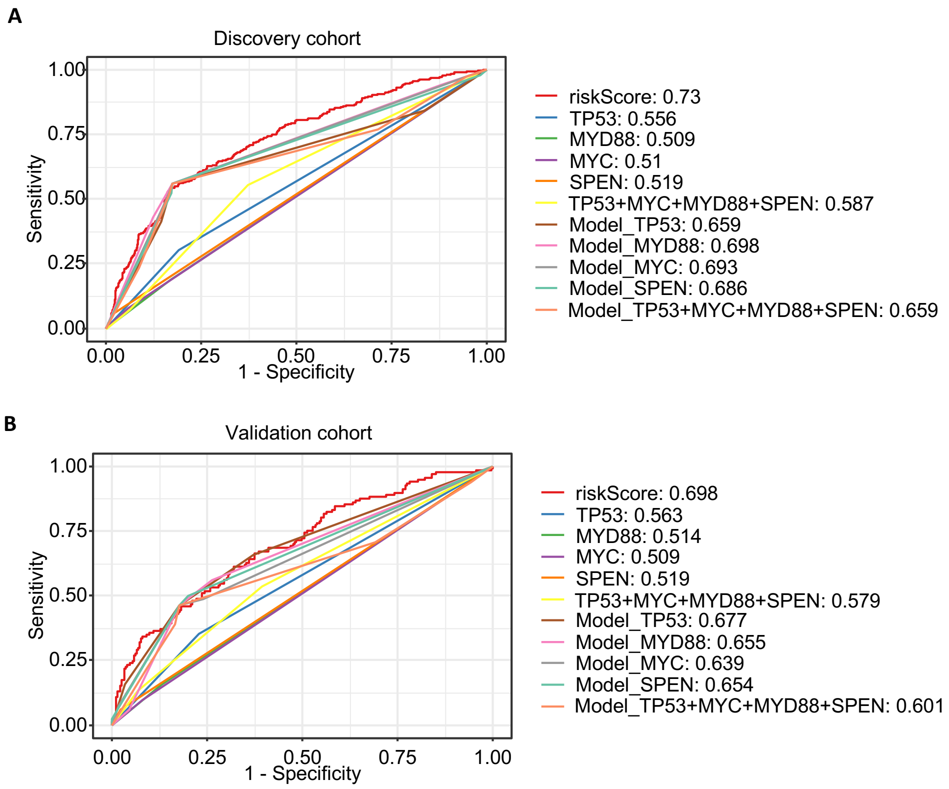

Figure S6. Evaluation of the 24-gene expression score with or without considering the mutational status of *TP53*, *MYD88*, *MYC*, and *SPEN* in predicting two-year outcomes in DLBCL patients.

Receiver operating characteristic (ROC) curves demonstrating the performance of the indicated factors in identifying DLBCL patients with two-year poor outcomes in the discovery cohort (**A**) and validation cohort (**B**). Numbers represent the AUC values. AUC, area under the curve. Model, 24-gene expression score.

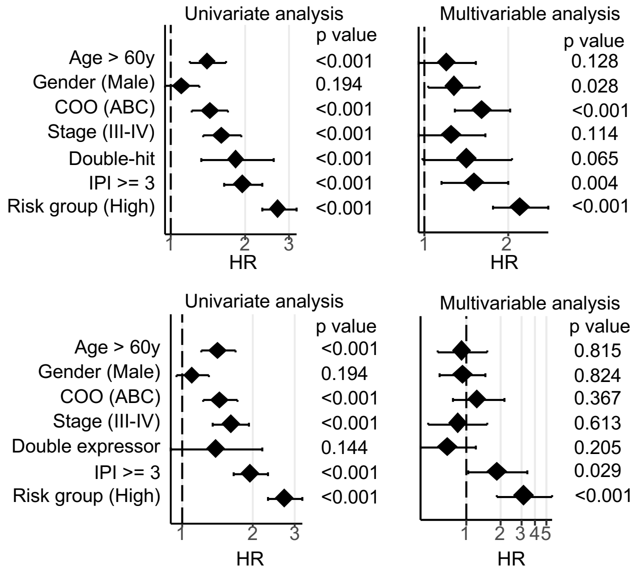

Figure S7. The independent prognostic performance of the 24-gene expression score.

Univariate and multivariable Cox regression was performed to assess the independent prognostic significance of the 24-gene expression score when considering double-hit and double-expressors in the analysis. The evaluation was conducted exclusively on DLBCL patients for whom both the 24-gene expression score and the status of double-hit and double-expressor (n=270) of *BCL2/MYC* were available. Among 1376 samples with 24-gene risk scores, 846 cases were evaluated for double-hit status, with 63 identified as double-hit. Double-expressor status was available only for those in the GSE117556 cohort. Of these, 270 patients had their double-expressor status accessed, with 92 identified as double-expressors. HR, hazard ratio. IPI, International Prognostic Index. COO, cell-of-origin.

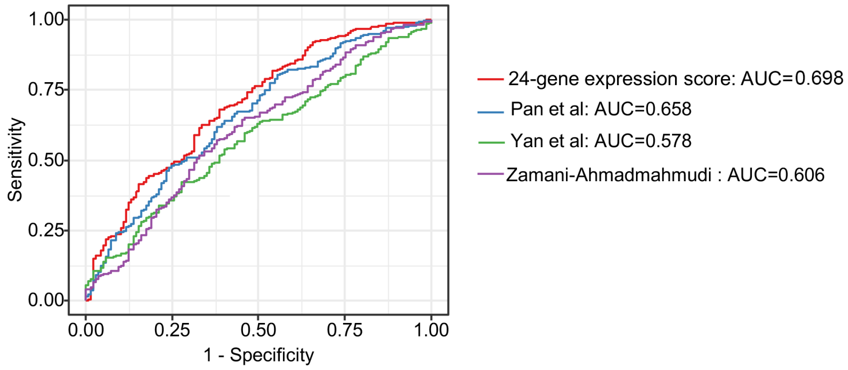

Figure S8. Comparison of the performance between the 24-gene expression score and other existing classifiers in predicting two-year outcomes in DLBCL patients.

ROC curves demonstrating the comparison of the performance of our 24-gene expression score and three other existing classifiers[14-16] in predicting two-year outcomes in the validation cohort (n=412). ROC, receiver operating characteristic; AUC, area under the curve.

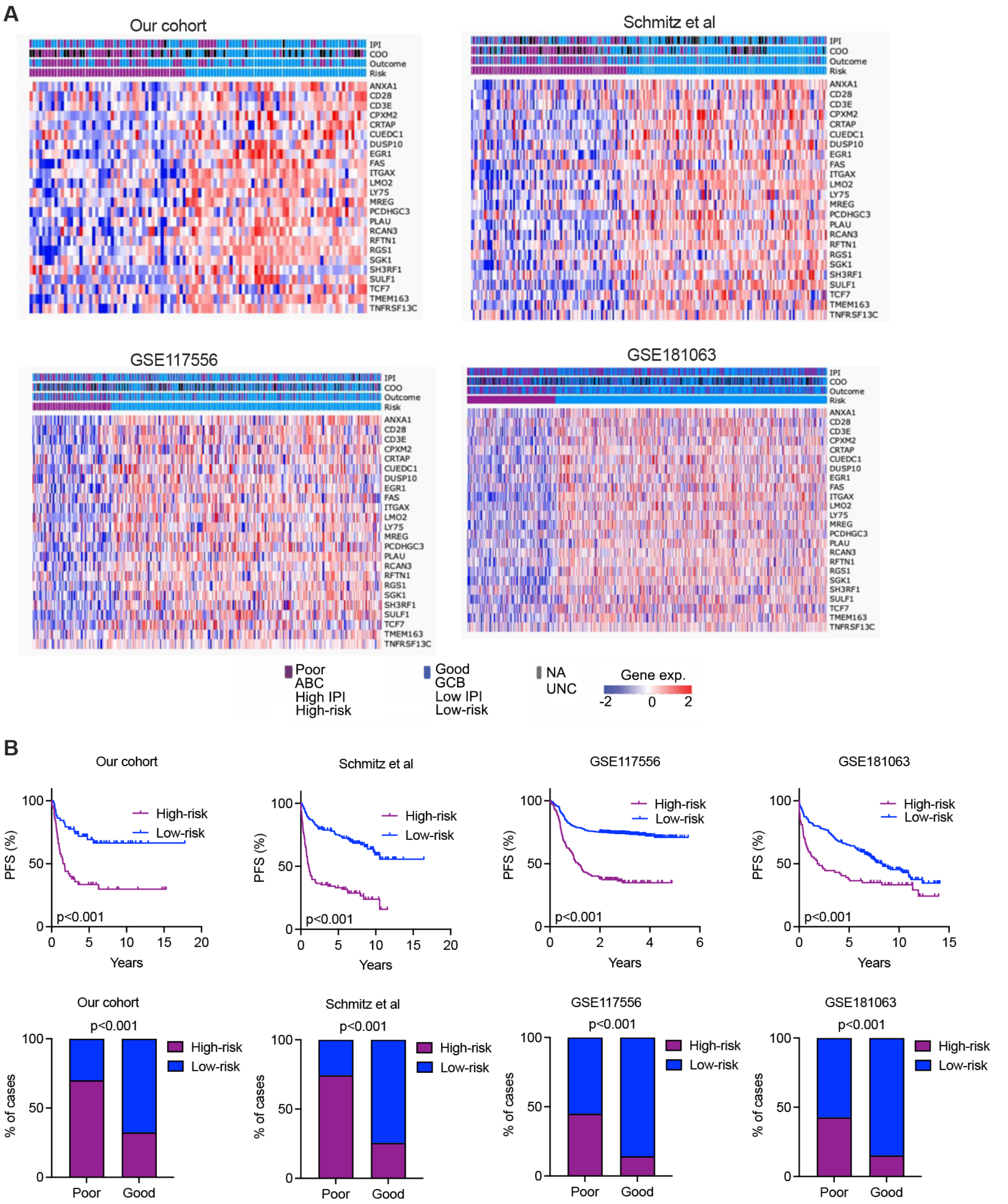

Figure S9. Comparison of high- and low-risk groups identified by the 24-gene expression scores across individual cohorts.

(**A**) Heat maps showing the expression levels of the 24 genes in DLBCL tumors in individual cohorts. Samples are represented as columns, ordered by risk groups. Within each group, samples were ordered following the input of the data. Each row represents a gene. (**B**) Kaplan–Meier survival analyses (top panel) illustrating the difference in PFS between high- and low-risk DLBCL patients across the indicated individual cohorts. Bar charts (bottom panel)

displaying the distribution of high- or low-risk DLBCL patients within the poor and good outcome groups for each cohort. PFS, progression-free survival. For the bar charts, the p value was calculated using Fisher's exact test, and for the Kaplan–Meier survival analyses, the p value was determined by the log-rank test.

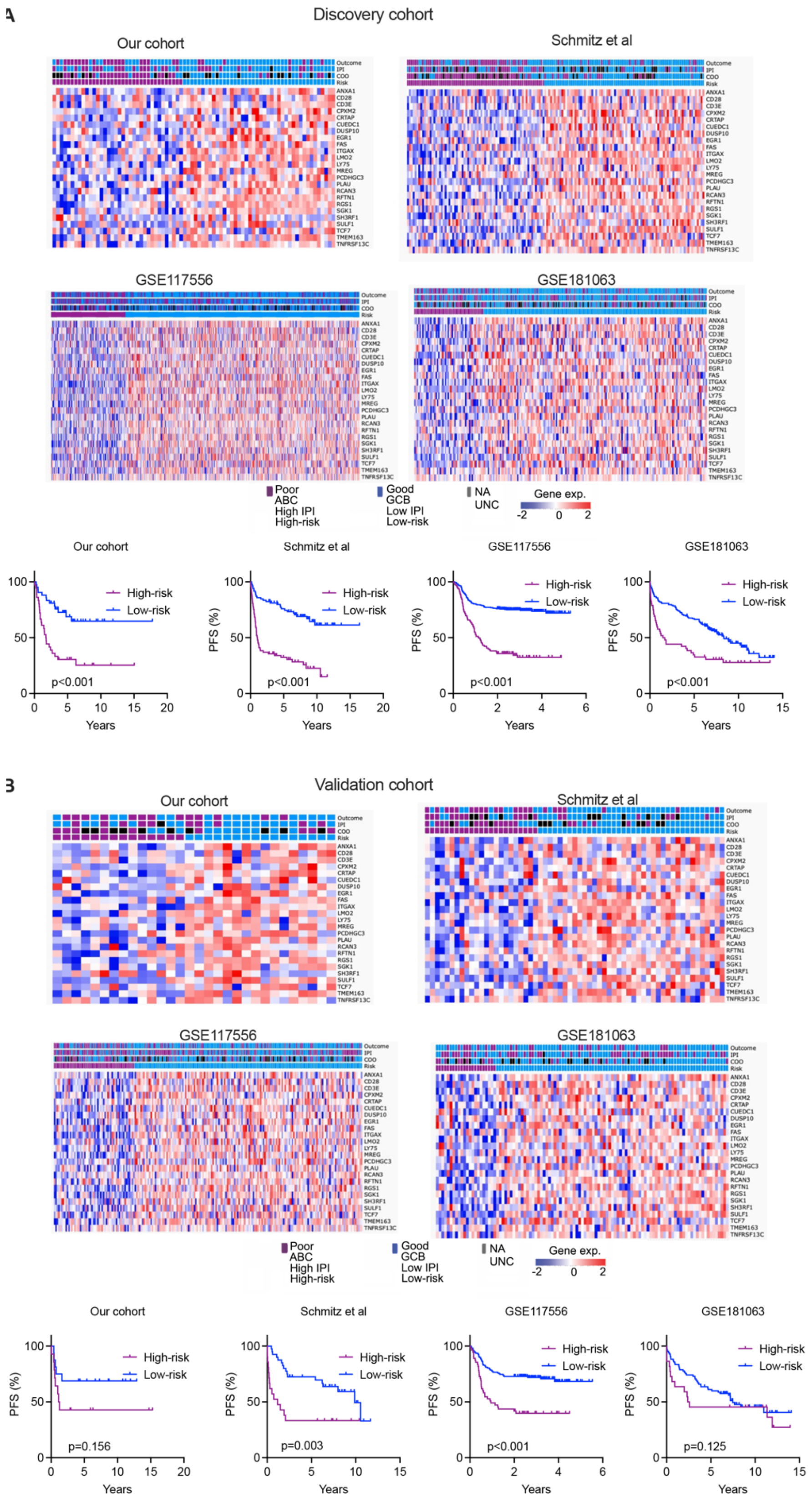

Figure S10. Comparison of high- and low-risk groups identified by the 24-gene expression scores in discovery and validation cohorts.

Top panel of **A** and **B**: Heat maps showing the expression levels of the 24 genes in DLBCL tumors in individual cohorts. Samples are represented as columns, ordered by risk groups. Within each group, samples were ordered following the input of the data. Each row represents a gene. The bottom panel of **A** and **B**: Kaplan–Meier survival analyses illustrating the difference in PFS between high- and low-risk DLBCL patients across the indicated individual cohorts. For the Kaplan–Meier survival analyses, the p value was determined by the log-rank test.

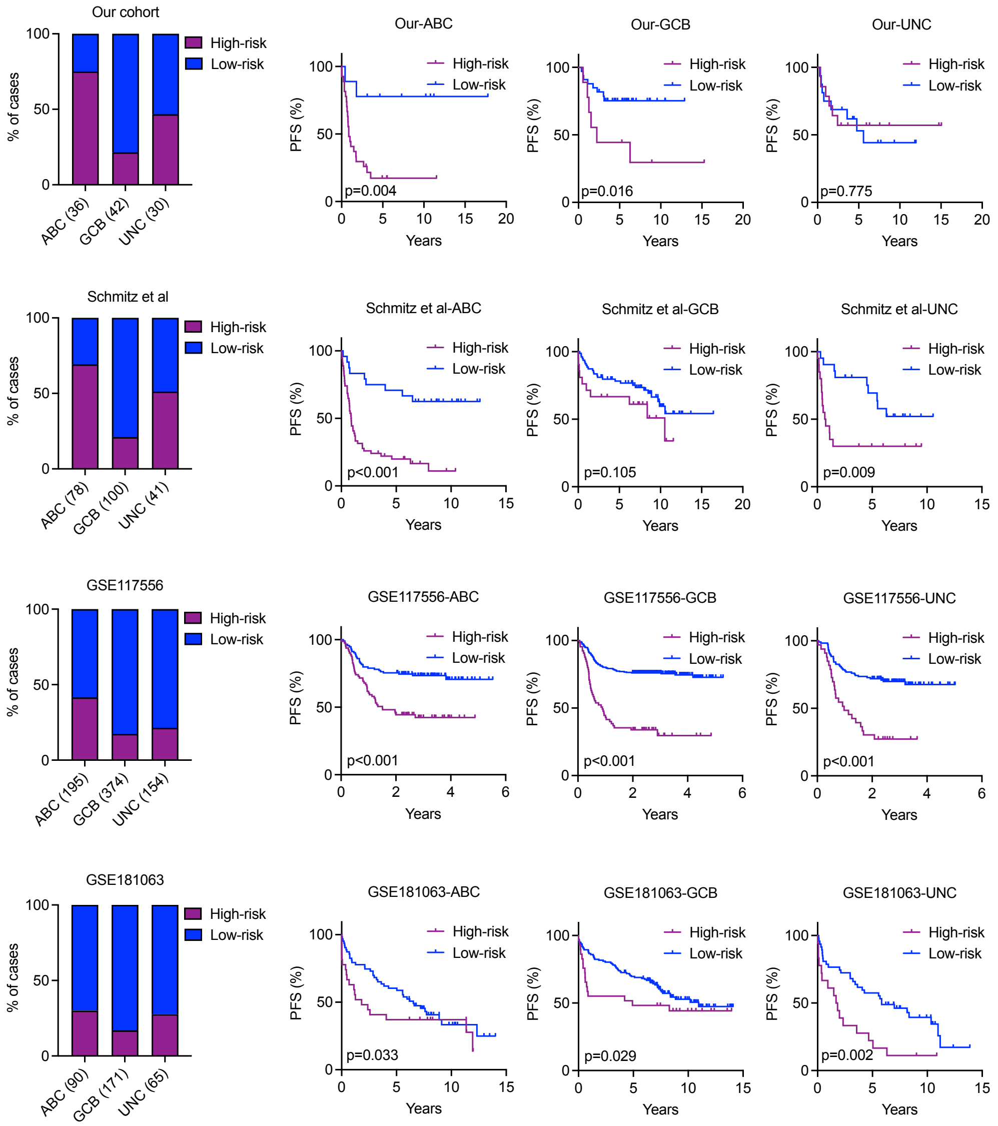

Figure S11. Risk stratification by 24-gene expression score across different COO subtypes in the individual cohorts.

The bar charts in the left panel show the distribution of high- and low-risk DLBCL patients across COO subtypes in the individual cohorts. The Kaplan–Meier survival analyses in the right panel illustrate the differences in PFS between high- and low-risk DLBCL patients in the indicated groups. PFS, progression-free survival. UNC, unclassified. For the Kaplan–Meier survival analyses, the *P* value was determined by the log-rank test.

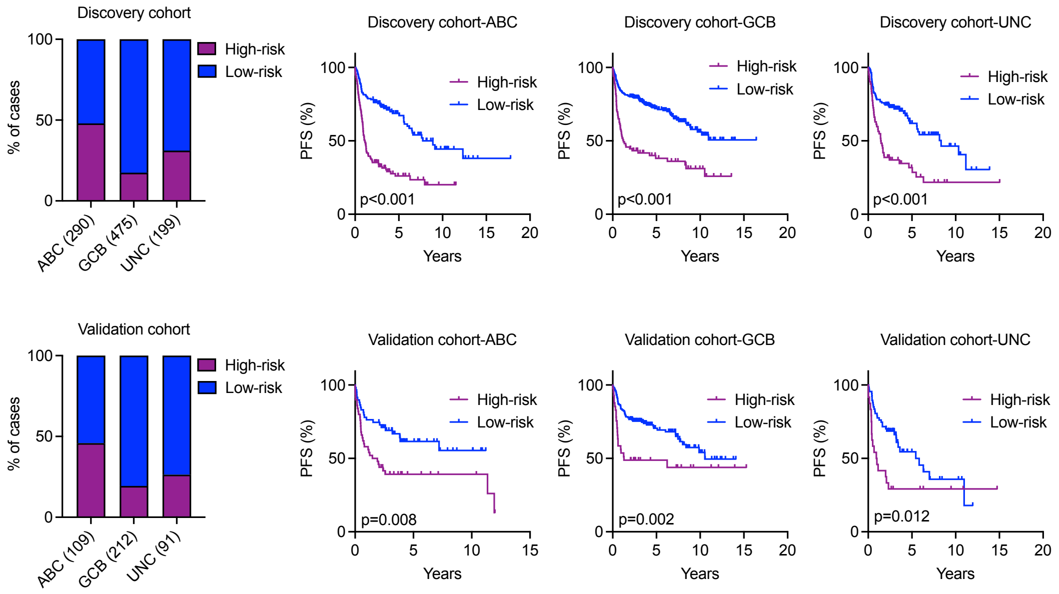

Figure S12. Risk stratification by 24-gene expression score across different COO subtypes in the discovery and validation cohorts.

The bar charts (left panel) show the distribution of high- and low-risk DLBCL patients among different COO subtypes. In the right panel, the Kaplan–Meier survival analyses illustrate the difference in PFS between high- and low-risk DLBCL patients within these COO subtypes in the discovery (n=964) cohort and validation cohort (n=412), respectively. PFS, progression-free survival; UNC, unclassified. For the Kaplan–Meier survival analyses, the *P* value was calculated by the log-rank test.

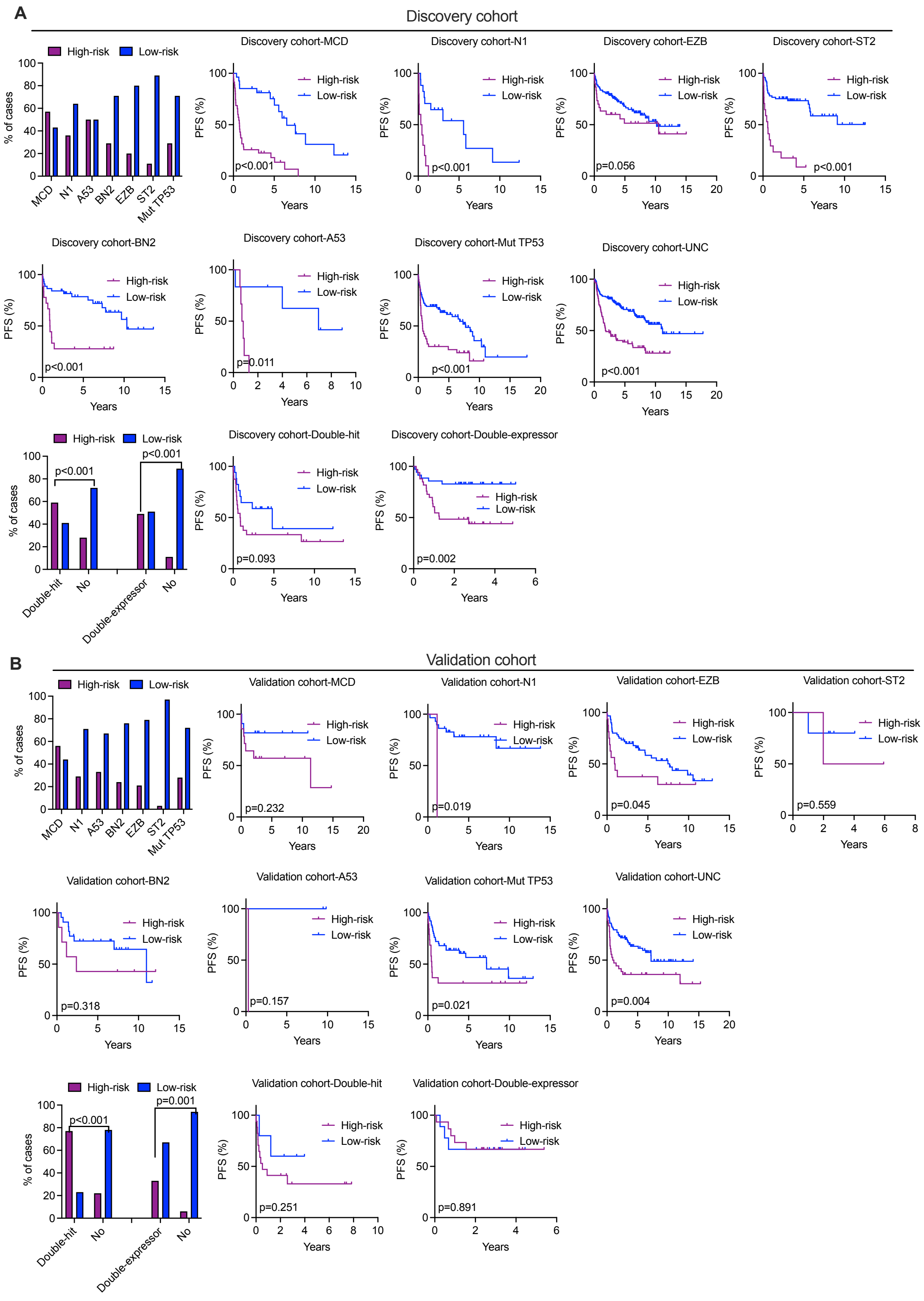

Figure S13. Risk stratification by 24-gene expression score across different DNA genetic subtypes in the discovery and validation cohorts.

(**A-B**) The bar charts and Kaplan–Meier survival analyses illustrating the distribution and the difference in PFS in high-risk and low-risk DLBCL patients in the indicated DNA clusters from the discovery cohort (**A**, n=964) and validation cohort (**B**, n=412), respectively. For bar charts, Fisher's exact test was used to calculate a p value. For the Kaplan–Meier survival analyses, the *P* value was calculated by the log-rank test.

References

1. Ren W, Ye X, Su H, Li W, Liu D, Pirmoradian M*, et al.* Genetic landscape of hepatitis B virus-associated diffuse large B-cell lymphoma. *Blood* 2018 Jun 14; **131**(24)**:** 2670-2681.

2. de Miranda NF, Georgiou K, Chen L, Wu C, Gao Z, Zaravinos A*, et al.* Exome sequencing reveals novel mutation targets in diffuse large B-cell lymphomas derived from Chinese patients. *Blood* 2014 Oct 16; **124**(16)**:** 2544-2553.

3. Georgiou K, Chen L, Berglund M, Ren W, de Miranda NF, Lisboa S*, et al.* Genetic basis of PD-L1 overexpression in diffuse large B-cell lymphomas. *Blood* 2016 Jun 16; **127**(24)**:** 3026-3034.

4. Ye X, Ren W, Liu D, Li X, Li W, Wang X*, et al.* Genome-wide mutational signatures revealed distinct developmental paths for human B cell lymphomas. *J Exp Med* 2021 Feb 1; **218**(2)**:** e20200573.

5. Li H, Durbin R. Fast and accurate short read alignment with Burrows-Wheeler transform. *Bioinformatics* 2009 Jul 15; **25**(14)**:** 1754-1760.

6. Koboldt DC, Chen K, Wylie T, Larson DE, McLellan MD, Mardis ER*, et al.* VarScan: variant detection in massively parallel sequencing of individual and pooled samples. *Bioinformatics* 2009 Sep 1; **25**(17)**:** 2283-2285.

7. Li R, Li Y, Fang X, Yang H, Wang J, Kristiansen K*, et al.* SNP detection for massively parallel whole-genome resequencing. *Genome Res* 2009 Jun; **19**(6)**:** 1124-1132.

8. Rimmer A, Phan H, Mathieson I, Iqbal Z, Twigg SRF, Consortium WGS*, et al.* Integrating mapping-, assembly- and haplotype-based approaches for calling variants in clinical sequencing applications. *Nat Genet* 2014 Aug; **46**(8)**:** 912-918.

9. Robinson JT, Thorvaldsdottir H, Winckler W, Guttman M, Lander ES, Getz G*, et al.* Integrative genomics viewer. *Nat Biotechnol* 2011 Jan; **29**(1)**:** 24-26.

10. Ritchie ME, Phipson B, Wu D, Hu Y, Law CW, Shi W*, et al.* limma powers differential expression analyses for RNA-sequencing and microarray studies. *Nucleic Acids Res* 2015 Apr 20; **43**(7)**:** e47.

11. Sherman BT, Hao M, Qiu J, Jiao X, Baseler MW, Lane HC*, et al.* DAVID: a web server for functional enrichment analysis and functional annotation of gene lists (2021 update). *Nucleic Acids Res* 2022 Jul 5; **50**(W1)**:** W216-W221.

12. Foltz SM, Greene CS, Taroni JN. Cross-platform normalization enables machine learning model training on microarray and RNA-seq data simultaneously. *Commun Biol* 2023 Feb 25; **6**(1)**:** 222.

13. Zhang T, Liu H, Jiao L, Zhang Z, He J, Li L*, et al.* Genetic characteristics involving the PD-1/PD-L1/L2 and CD73/A2aR axes and the immunosuppressive microenvironment in DLBCL. *J Immunother Cancer* 2022 Apr; **10**(4).

14. Pan M, Yang P, Wang F, Luo X, Li B, Ding Y*, et al.* Whole transcriptome data analysis reveals prognostic signature genes for overall survival prediction in diffuse large B cell lymphoma. *Front Genet* 2021; **12:** 648800.

15. Yan J, Yuan W, Zhang J, Li L, Zhang L, Zhang X*, et al.* Identification and validation of a prognostic prediction model in diffuse large B-cell lymphoma. *Front Endocrinol (Lausanne)* 2022; **13:** 846357.

16. Zamani-Ahmadmahmudi M, Nassiri SM. Development of a Reproducible Prognostic Gene Signature to Predict the Clinical Outcome in Patients with Diffuse Large B-Cell Lymphoma. *Sci Rep* 2019 Aug 21; **9**(1)**:** 12198.
